# The Safety and Efficacy of Nanosecond Pulsed Electric Field in Patients With Hepatocellular Carcinoma: A Prospective Phase 1 Clinical Study Protocol

**DOI:** 10.3389/fonc.2022.869316

**Published:** 2022-07-13

**Authors:** Min Xu, Danxia Xu, Gang Dong, Zhigang Ren, Wu Zhang, Tuerganaili Aji, Qiyu Zhao, Xinhua Chen, Tian’an Jiang

**Affiliations:** ^1^ Department of Ultrasound Medicine, The First Affiliated Hospital, School of Medicine, Zhejiang University, Hangzhou, China; ^2^ Key Laboratory of Pulsed Power Translational Medicine of Zhejiang Province, Hangzhou, China; ^3^ Department of Ultrasound, The First Affiliated Hospital of Zhengzhou University, Zhengzhou, China; ^4^ Department of Infectious Diseases, The First Affiliated Hospital of Zhengzhou University, Zhengzhou, China; ^5^ Shulan International Medical College, Shulan (Hangzhou) Hospital Affiliated to Zhejiang Shuren University, Hangzhou, China; ^6^ Department of Surgery, The First Affiliated Hospital of Xinjiang Medical University, Urumqi, China; ^7^ Department of Hepatobiliary and Pancreatic Surgery, The First Affiliated Hospital, School of Medicine, Zhejiang University, Hangzhou, China

**Keywords:** nanosecond pulsed electric field (nsPEF), irreversible electroporation (IRE), hepatocellular carcinoma (HCC), ablation, protocol

## Abstract

**Background:**

Hepatocellular carcinoma (HCC) is a highly aggressive malignancy. Irreversible electroporation (IRE) is an ablative modality that uses high-voltage electrical pulses to permeabilize the cell membrane leading to cell necrosis. Unlike traditional thermal ablation, IRE is hardly affected by the “heat-sink” effect and can prevent damage of the adjacent vital structures. Nanosecond pulsed electric field (nsPEF) is a new IRE technique using ultra-short pulses (nanosecond duration), can not only penetrate the cell membranes, but also act on the organelles. Sufficient preclinical researches have shown that nsPEF can eliminate HCC without damaging vital organs, and elicit potent anti-tumor immune response.

**Objective:**

This is the first clinical study to evaluate feasibility, efficacy, and safety of nsPEF for the treatment of HCC, where thermal ablation is unsuitable due to proximity to critical structures.

**Methods and analysis:**

We will conduct an open-labeled, single-arm, prospective, multicenter, and objective performance criteria trial. One hundred and ninety-two patients with HCC, in which the tumor is located immediately (<0.5 cm) adjacent to the portal vein, hepatic veins, bile duct, gastrointestinal tract, or diaphragm, will be enrolled among 4 academic medical centers. The primary outcomes are the rate of complete ablation at 1 month and adverse events. Secondary outcomes include technical success, technique efficacy, nsPEF procedural characteristics, local tumor progression, and local progression-free survival.

**Ethics and dissemination:**

The trial will be conducted according to the ethical principles of the Declaration of Helsinki and has been approved by the ethics committee of all participating centers. The results of this study will be published in peer-reviewed scientific journals and presented at relevant academic conferences.

**Conclusions:**

This study is the Phase 1 clinical trial to evaluate the efficacy and safety of nsPEF in patients with HCC at high-risk locations where thermal ablation is contra-indicated. The results may expand the options and offer an alternative therapy for HCC.

**Clinical Trial Registration:**

ClinicalTrials.gov, identifier NCT04309747.

## 1 Introduction

Liver cancer is a major global health challenge that is predicted to affect more than 1 million individuals annually by 2025. Hepatocellular carcinoma (HCC) is the most common type of primary liver cancer (90%) and the fourth leading cause of cancer-related deaths worldwide (1). Local ablation is considered a potentially curative therapy for small HCC, as are surgical resection and liver transplantation (2, 3). Most ablation techniques, effected through radiofrequency, microwave, or laser, are based on thermal changes of the ablated tissue. However, many tumors cannot be treated with thermal ablation owing to hazardous tumor locations, and thermal damage to adjacent non-targeted structures can result in serious complications, such as hemorrhage, biliary fistula or intestinal perforation. Moreover, heat drawn away from the targeted tumor when it is adjacent to vessels, i.e., the heat sink effect, can result in incomplete ablation (4–6).

Irreversible electroporation (IRE) is a non-thermal ablation modality that has been advocated for solid hepato-pancreatico-biliary tumors. IRE delivers high-voltage electric pulses to permeabilize the cell membrane and consequently cause cell death, mostly by apoptosis (7, 8). Unlike thermal ablation, with IRE the extracellular connective tissue stays intact. This enables ablating tumors that are close to or involving vital structures such as the portal vein, hepatic veins, or bile duct. In addition, treatment efficacy of IRE is not impeded by heat sink effect (9–11). Based on these specific properties, IRE should be considered an ideal tool for patients for whom thermal ablation is too aggressive (12, 13).

**Figure 1:**
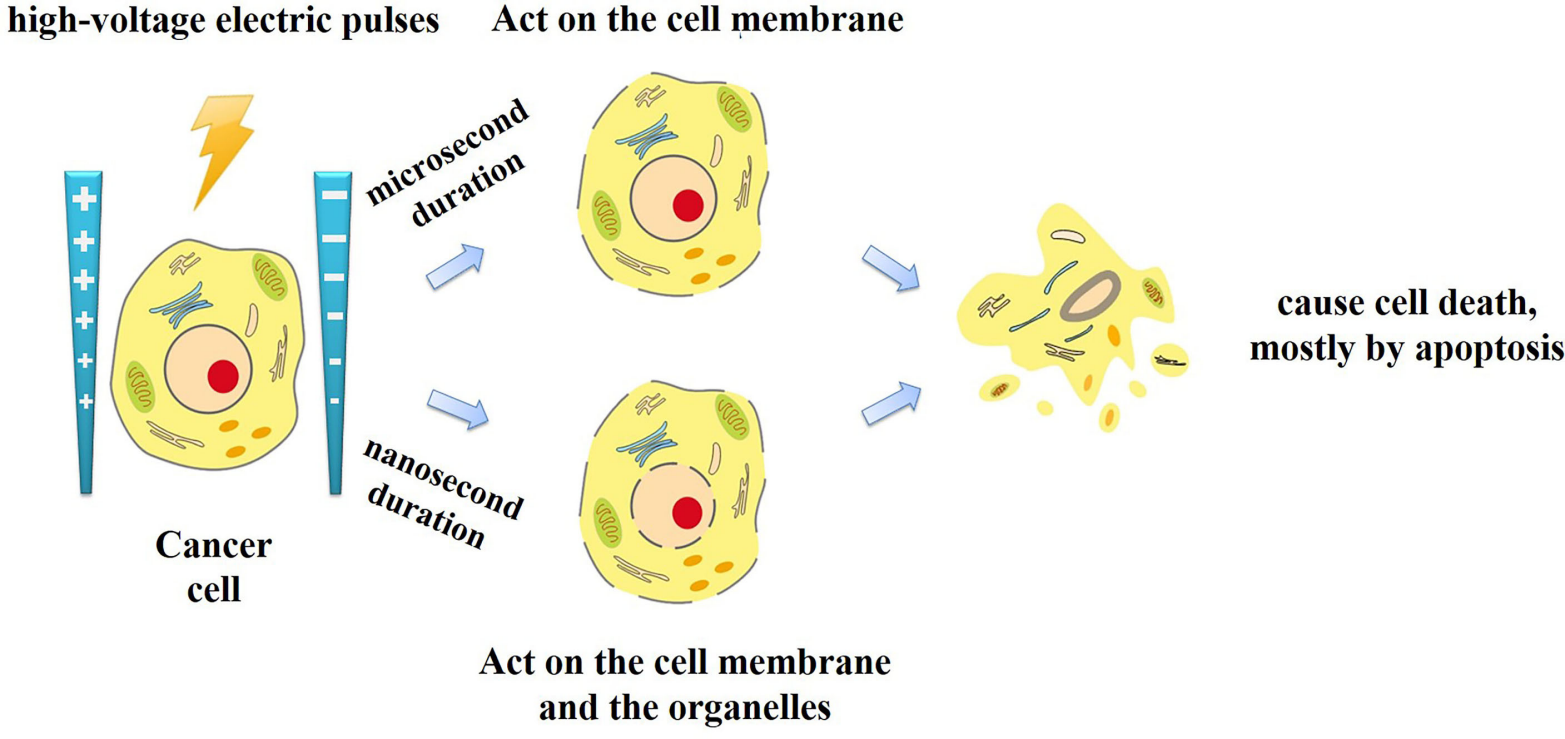
The mechanisms of high-voltage electric pulses with microsecond or nanosecond duration.

Traditional IRE applies electric pulses of microsecond duration. Recently, a new type of IRE technique has been introduced, called nanosecond pulsed electric field (nsPEF). Unlike traditional microsecond duration IRE, the nsPEF with the duration from a few nanoseconds to hundreds of nanoseconds and the amplitude from 10 kV/cm to 300 kV/cm has been applied for tumor ablation. Since the duration shorter than the charging time constant of the cell membrane, nsPEF can not only penetrate the cell membranes, but also act on the organelles such as the endoplasmic reticulum, mitochondria, and nucleus (**Figure 1**). Cell responses to nsPEF include calcium mobilization (14), cytoskeleton destruction (15), activation of signaling pathways (16), and induction of apoptosis (17). Moreover,treatment with microsecond pulsed electric field has the undesirable side effect of adjacent skeletal muscle stimulation. Relatively long exposure of the skeletal muscle or its motor nerves to a high exogenous electric field will depolarize the cells and cause intense contractions. Previous studies found that shortening the time of the electric field exposure from microseconds to nanoseconds can reduce pain and involuntary muscle contraction (18). Therefore, different interaction mechanisms with traditional IRE, nsPEF expands the options and offers new opportunities for oncological therapy (**Table 1**).

**Table 1:** Comparison of the mechanism of nsPEF and thermal ablation.

	nsPEF	RFA	MWA	Cryoablation
Fundamental principles	Utilizing high-frequency electric pulses penetrate the cell membranes, and act on the organelles	Utilizing high-frequency alternating current to generate heat	Utilizing electromagnetic waves to generate heat	Utilizing liquefied gases to induce the freezing–thawing cycle of targeted lesions
Treatment temperature	Nonthermal	60–100°C	>100°C	< – 40°C
Mechanism of tumor cell injury	Mainly apoptosis	Mainly necrosis	Mainly necrosis	Mainly necrosis
Advantages	Limited risk of thermal injury to neighbouring critical structures Unsensitive to heat sink effect	Well evaluated treatment	Higher and faster temperature picks reached than with RFA (less sensitive to heat sink effect than RFA)	Easy monitoring with imaging of ice ball progression
Limitations	General anesthesia Muscle contraction	Thermal injury of adjacent structure Heat sink effect	No reliable end point to set the amount of energy deposition	Cryoshock with first device

Killing cancer cells *in vitro via* nsPEF has been extensively explored, and successful tumor ablation has been demonstrated in various animal models, including malignant melanoma, skin basal cell carcinoma, lung squamous cell cancer and pancreatic cancer (17, 19–22). Most importantly, a clinical trial showed that nsPEF could successfully treat basal cell carcinoma in human patients, with significant efficacy and minimal invasion (23).

For the treatment of liver cancer, nsPEF has shown promising therapeutic prospects in both cell and animal experiments (**Supplementary Appendix 1**). Our preclinical studies have verified the efficacy of nsPEF, that is, that nsPEF can lead to long-term disease-free survival without recurrence (24, 25). In addition to inducing cell death, nsPEF inhibited cell proliferation and angiogenesis in tumors, triggered an immune response, and prevented secondary tumor growth (26–29).

Here, we propose to conduct the first-in-human trial to evaluate the efficacy and safety of nsPEF in patients with localized HCC, for whom thermal ablation is considered unsuitable.

## 2 Methods and Analysis

### 2.1 Study Design

**Figure 2:**
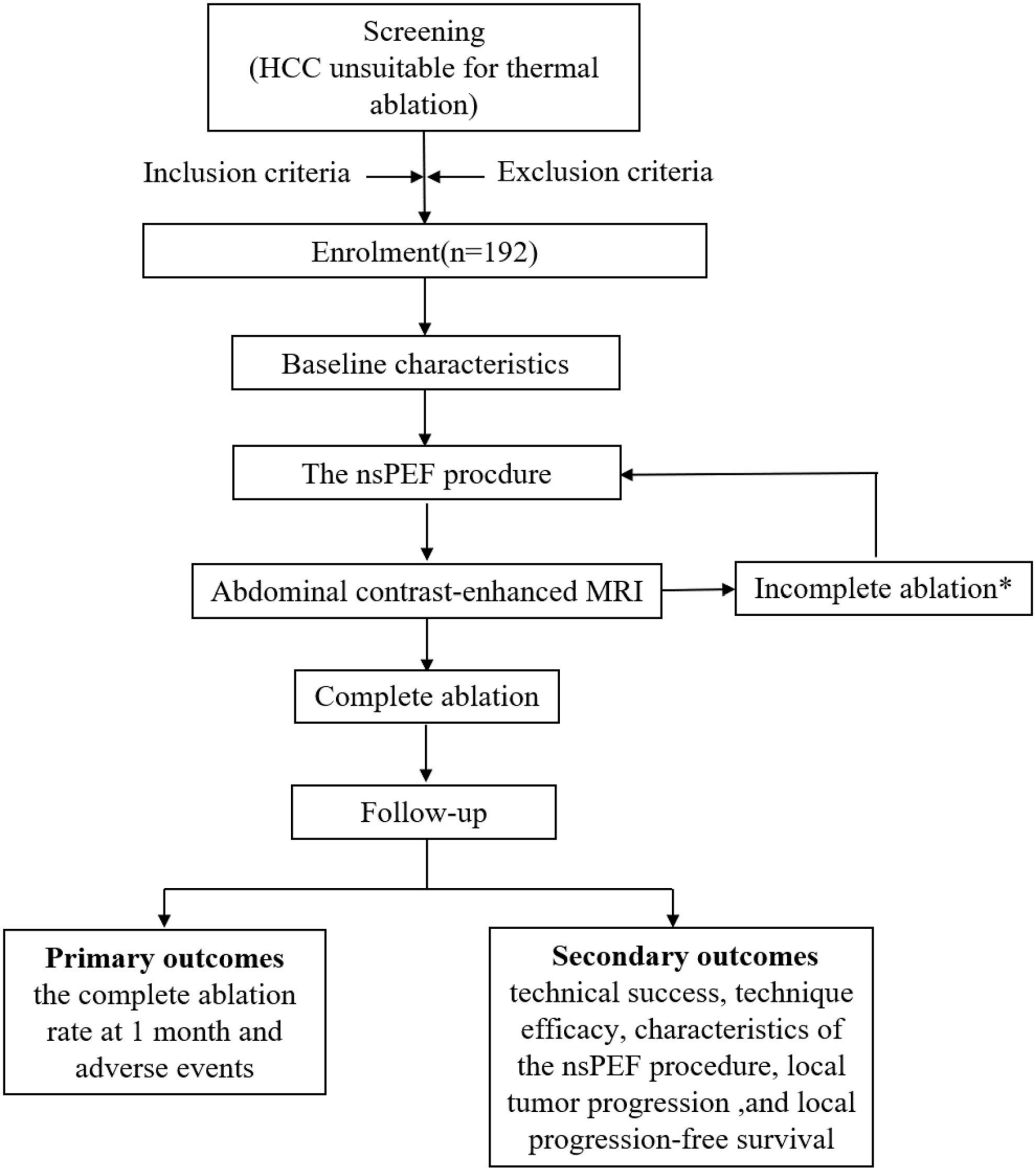
Flow chart of study. *Contrast-enhanced MRI will be performed 1 month after the procedures to evaluate technique efficacy. In the event of incomplete ablation, an additional nsPEF procedure will be conducted with the same technique. If the residual tumor is still viable after the second session, then nsPEF is considered failed, and the patient will be excluded from the trial and referred to other therapies. HCC, hepatocellular carcinoma; nsPEF, nanosecond pulsed electric field; MRI, magnetic resonance imaging.

This open-labeled, single-arm, prospective, multicenter trial will be conducted in 4 academic medical centers in China, as follows: First Affiliated Hospital, School of Medicine, Zhejiang University, Hangzhou; First Affiliated Hospital of Zhengzhou University, Zhengzhou; Shulan (Hangzhou) Hospital, Hangzhou; and First Affiliated Hospital of Xinjiang Medical University, Xinjiang. This trial has been approved by the committee for medical and health ethics of all the centers and registered on ClinicalTrials.gov (NCT04309747). The trial protocol is in accordance with all the recommendations of the SPIRIT (Standard Protocol Items: Recommendations for Interventional Trials) 2013 statement (30). Written informed consent will be obtained from all participants prior to enrollment. The flow diagram of the study is presented in **Figure 2**.

### 2.2 Patient Population

Potential trial participants will be identified at an institutional multidisciplinary tumor board, comprised of interventional radiologists, hepatobiliary surgeons, medical oncologist, and diagnostic radiologists. The potential subjects will subsequently be referred for enrollment and eligibility screening. All candidates will be reviewed for safety and eligibility by an experienced interventional radiologist, participating in the research project at each center. All eligible patients will be explained about the study protocol in details. Only those who sign the consent form will enter the trial. The inclusion and exclusion criteria are listed in **Table 2**.

**Table 2:** Patients inclusion and exclusion criteria.

Inclusion criteria	Exclusion criteria
HCC diagnosed histologically or clinically according to the guidelines of the American Association for the Study of Liver Diseases tumors located immediately (<0.5 cm) adjacent to the portal vein, hepatic veins, bile duct, gastrointestinal tract, or diaphragm age ≥ 18 years no evidence of extrahepatic metastasis single tumor with a maximum diameter of ≤5cm, or the number of tumors of ≤3 and a maximum diameter of ≤3cm. Child-Pugh A or B Performance status according to Eastern Cooperative Oncology Group (ECOG) ≤2. written informed consent	Ventricular cardiac arrhythmia Congestive heart failure, NYHA Class ≥ 3 Active coronary artery disease History of epilepsy Any implanted stimulation device Other treatment <6 weeks prior to treatment presence of vascular invasion or extrahepatic metastasis. Severe coagulation abnormalities

### 2.3 Baseline Characteristics

Standard evaluation of the patient before the nsPEF procedure should include a general health history review (demographics, past medical history, allergies, and medications), assessment of performance status and pain, general anesthetic review, electrocardiogram, echocardiography, and laboratory tests (blood routine examination, blood coagulation, liver function, tumor marker, and myocardial enzyme). All included patients will have a pre-intervention radiological assessment within 4 weeks before the nsPEF procedure, including abdominal contrast-enhanced magnetic resonance imaging (MRI), CT angiography of the liver, chest computed tomography (CT), contrast-enhanced ultrasound (CEUS) of the liver, and abdominal contrast-enhanced CT when MRI is contraindicated.

### 2.4 Interventions

The nsPEF procedure will be performed under general anesthesia with endotracheal intubation. Intraoperative nondepolarizing neuromuscular blocking agents (rocuronium or cis-atracurium) will be applied in combination to achieve deep muscle relaxation, in which no muscle tremor visible to the naked eye or a train of four stimulations (TOF) is 0. Deep muscle relaxation can eliminate the muscle contractions caused by the high-voltage pulsed electric fields and reduce the injuries of the target organs due to electrode displacements.

**Figure 3:**
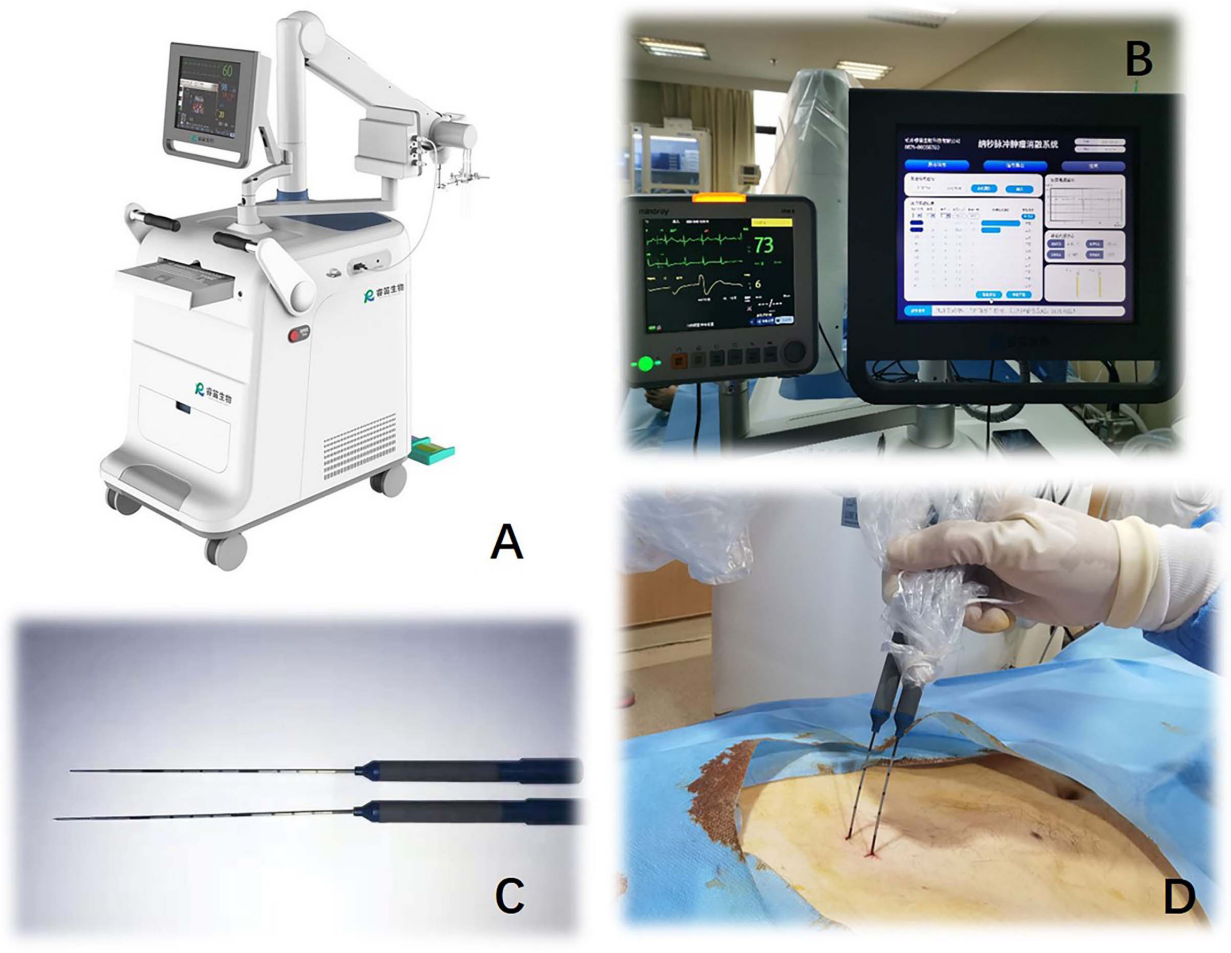
Nanosecond pulsed electric field equipment (Hangzhou Ruidi Biotech Ltd, Hangzhou, Zhejiang, China) **(A)** console. **(B)** displayer. **(C, D)** 19G monopolar needle electrodes.

**Figure 4:**
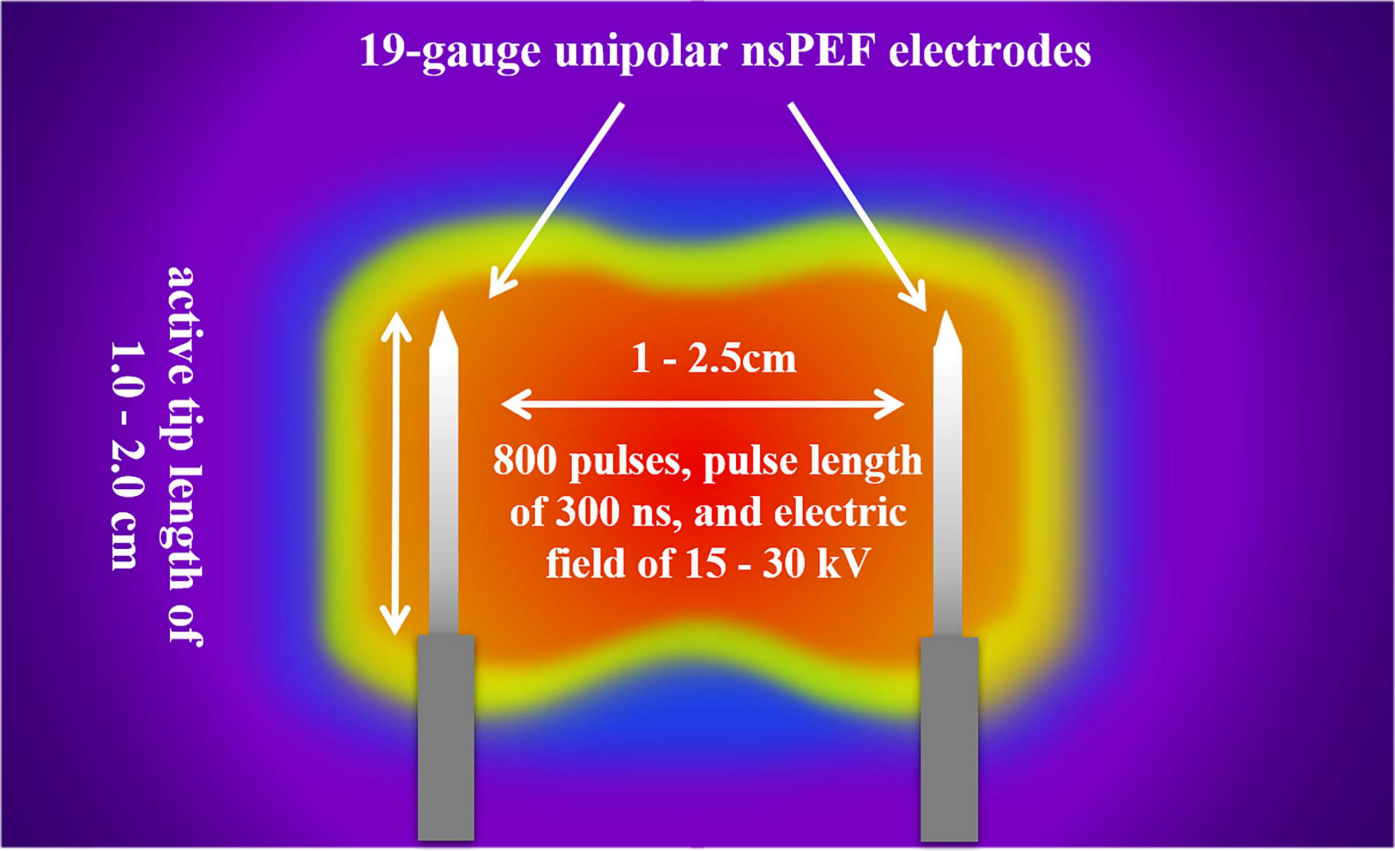
The nsPEF procedure strategy. Depending on the individual tumor size and shape, 2 to 6 19-gauge unipolar electrodes with an appropriate active tip length of 1.0 to 2.0 cm will be placed 1.0 to 2.5 cm apart using ultrasound guidance. The nsPEF will be conducted with 800 pulses of 300ns duration, and an electric field of 15 to 30 kV. nsPEF, nanosecond pulsed electric field.

The nsPEF procedure will be guided by CT or US. The nsPEF therapeutic apparatus manufactured by the Hangzhou Ruidi Biotech Ltd company (Hangzhou, Zhejiang, China) (**Figure 3**) will be used in this study. All electrodes will be placed parallel by trained interventional radiologists with extensive experience in percutaneous thermal ablation (via radiofrequency, microwave, or laser) and nonthermal ablation (IRE). Depending on the individual tumor size and shape, 2 to 6 19-gauge unipolar nsPEF electrodes with an appropriate active tip length of 1.0 to 2.0 cm will be placed 1.0 to 2.5 cm apart using ultrasound guidance. The nsPEF device generates 15 to 30 kV pulses, and voltage will be determined by a standard algorithm that uses factors such as the intended size of the ablation zone, the number of electrodes, the distance between electrodes, and the length of the active electrode tip (**Figure 4**). Once the electrodes are correctly placed, a test pulse at 5 kV will be delivered. After the test pulse confirms adequate conductivity, nsPEF will be conducted with 800 pulses, a pulse length of 300 ns, with electrocardiographic triggering.

**Figure 5:**
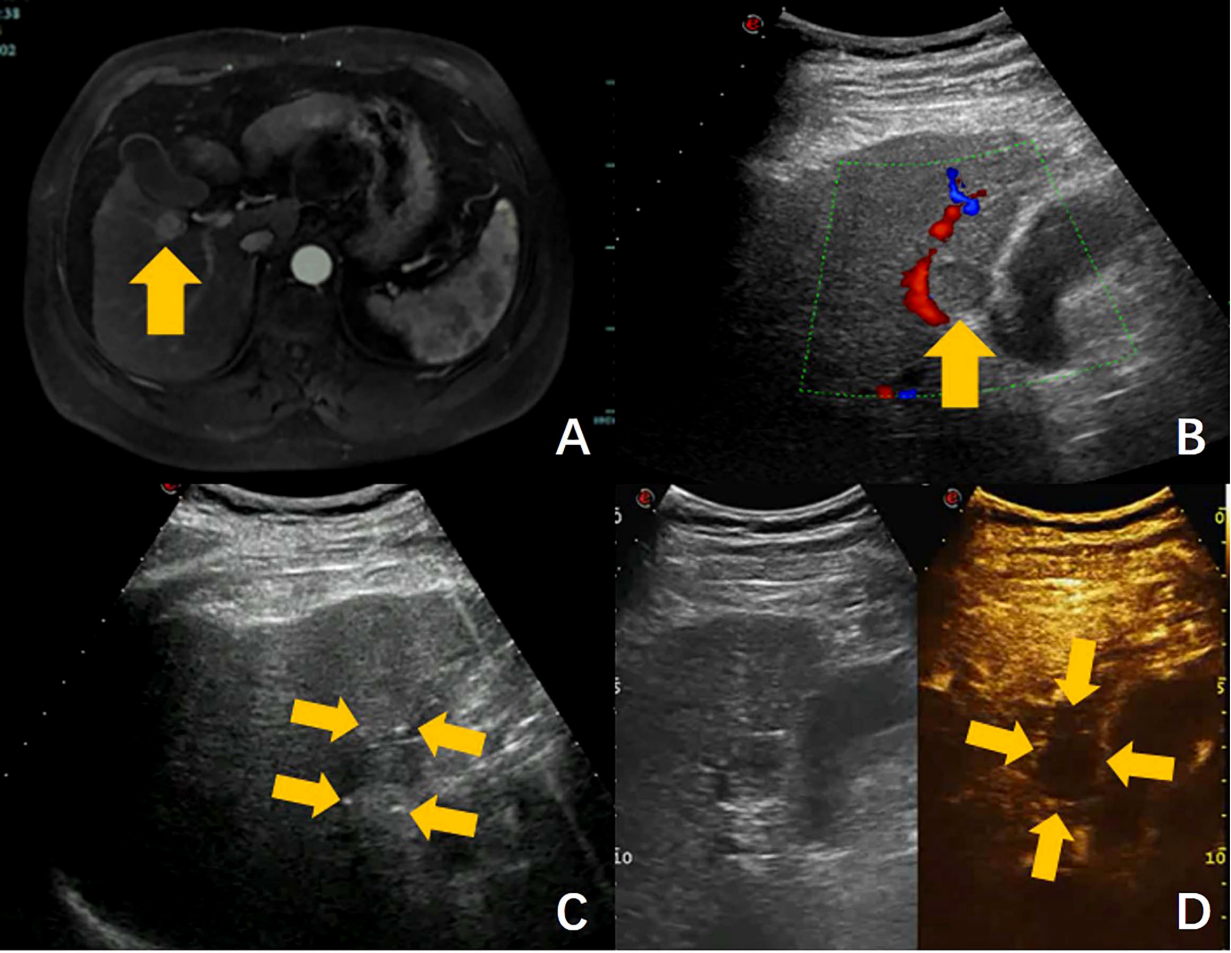
Ultrasound-guided nsPEF procedure for HCC adjacent to gallbladder. **(A)** Pre-ablation contrast-enhanced MRI revealed a hepatocellular carcinoma adjacent to gallbladder. **(B)** Ultrasound showed a hypoecho tumor located in close contact with gallbladder and right portal branch (arrow). **(C)** Ultrasound-guided nsPEF with a two-electrode configuration, and the head and tail end of the active tip of the electrodes can have punctuated enhancements (arrow). **(D)** The contrast-enhanced ultrasound image revealed no enhancement after ablation. (arrow).

After completion of the pulse applications, CEUS will be performed to confirm sufficient ablation, which is defined as an ablation zone that includes the entire target tumor and a safety margin of at least 0.5 cm. If the extent of the ablation zone is suspected insufficient, additional cycles of energy depositions for overlapped ablations will be performed, preferably after electrodes pullbacks (from 1 to 2 cm partial withdrawal of needles along the axis of the initial puncture) and/or partial or complete electrodes reinsertion (in different axis from initial puncture). A case is shown in **Figure 5** and **Video 1**.

### 2.5 Postprocedural Follow-Up

After the procedure, all patients will be monitored for at least another day in inpatient department, in accordance with current medical practice. Routine laboratory studies, electrocardiogram, and CEUS will be performed to assess procedure-related complications.

Contrast-enhanced MRI will be performed 1 month after the procedures to evaluate technique efficacy. Complete ablation is considered complete nonenhancement of the treated tumor, and incomplete ablation is the presence of residual tumor on contrast-enhanced MRI. In the event of incomplete ablation, an additional nsPEF procedure will be conducted with the same technique. If the residual tumor is still viable after the second session, then nsPEF is considered failed, and the patient will be excluded from the trial and referred to other therapies.

If efficacy is confirmed, then clinical, biological, and radiological examinations are required every 3 months during the first year, and once every 6 months thereafter. Baseline and follow-up images will be interpreted independently by all experienced diagnostic abdominal radiologists. At the end of the trial, 2 independent radiologists will review all images before and after treatment, and reach a consensus.

### 2.6 Study Outcomes

Treatment outcomes are defined in accordance with the Ahmed et al. (31, 32) proposal for standardization of terms and reporting criteria for imaging-guided ablation.

#### 2.6.1 Primary Outcomes

The primary outcomes are the complete ablation rate at 1 month, and adverse events. The later may be device-related, intraprocedural, postprocedural, or late, and will be reported using the NCI Common Terminology Criteria for Adverse Events (CTCAE) (**Supplementary Appendix 2**).

#### 2.6.2 Secondary Outcomes

The secondary outcomes are the following: technical success, technique efficacy, characteristics of the nsPEF procedure, local tumor progression (LTP), and local progression-free survival (LPFS). Specifically, technical success addresses whether the tumor is treated according to protocol and covered completely by the ablation zone; tumor coverage will be assessed immediately after the nsPEF procedure by CEUS.

Technique efficacy is regarded as radiologic complete ablation achieved after as many as 2 iterative nsPEF procedures. The nsPEF procedure characteristics include the following: number of electrodes used; active tip length of the electrodes; distance between electrodes; rate of pullback applications; total number of pulses delivered per procedure; total procedural time; ablation time; ablation volume; and sufficient ablative margin.

LTP describes the reappearance of HCC adjacent to the ablated zone after successful treatment. LPFS is defined as the time from the commencement date of the nsPEF procedure to the date of local progression. LPFS will be censored at the date of the last follow-up, when the patient has no evidence of local progression.

### 2.7 Calculation of Sample Size

Because this is a pilot study, there is currently no data on the complete ablation rate of nsPEF for the treatment of HCC. Previous studies showed a 77.3%-86% rate of complete ablation after the first IRE (with a traditional microsecond duration) in patients with HCC that was not appropriate for thermal ablation (11, 33). Our hypothesis is that 80% of tumors treated by nsPEF will achieve complete ablation by the 1-month follow-up. For the single arm OPC hypothesis, employing 80% power and with a 2.5% one-sided α, then 160 patients will be required. Assuming a 20% rate of withdrawal or loss during follow-up, 192 participants should be enrolled.

### 2.8 Statistical Methods

Continuous data will be expressed as median and range. Categorical variables will be shown as frequency and proportion. Survival curves and cumulative incidence of LTP will be generated using the Kaplan-Meier method. The Cox proportional hazards method will be used for univariate and multivariate analyses to determine prognostic factors. Technical success, technique efficacy, nsPEF procedural characteristics, and local tumor progression will be analyzed per tumor. The rate of adverse events and local progression-free survival will be analyzed per patient. *P* values < 0.05 will be considered statistically significant. All statistical analyses will be performed with a software package (SPSS, version 23.0; SPSS, Chicago, Ill).

### 2.9 Adverse Events

Device-related, periprocedural, and postprocedural adverse events will be measured using the CTCAE. Any serious adverse events will be documented in the medical records as well as in the electronic case report form and reported to the institutional review board by the responsible investigator, in accordance with the local regulations.

### 2.10 Data and Safety Monitoring Board

The DSMB will act in an independent, expert, and advisory capacity to monitor participant safety, and assess the efficacy and overall conduct of the study. The responsibilities of the DSMB are to monitor safety and efficacy data to guide recommendation for continuation of the study or early termination, and to evaluate the overall conduct of the trial. These responsibilities include monitoring: planned sample size assumptions; compliance with the protocol; recruitment figures and losses to follow-up; and continuing appropriateness of patient information. In addition, the DSMB’s responsibilities include reports on data quality and completeness of data.

### 2.11 Ethics and Dissemination

This study is conducted in accordance with the principles of the Declaration of Helsinki. The protocol has been approved by the ethics committee of all participating centers. Informed consent will be obtained from each participating patient in oral and written form. The results of this trial will be disseminated through peer-reviewed publications and conferences. Data will be available upon reasonable request.

## 3 Discussion

IRE is primarily a nonthermal ablation technique. The working mechanism of IRE is direct injury caused by high voltage electrical pulses, rather than thermal energy. The major advantage of IRE is its ability to preserve sensitive structures, which is not true of other ablative techniques such as radiofrequency ablation or microwave ablation. Previous clinical trials of IRE revealed encouraging results for the treatment of tumors that are close to major vessels or bile ducts, including those in the liver, pancreas, and kidney (34–36).

With recent developments in electrical engineering technology, IRE devices have gained nanosecond-duration pulses (nsPEF). The charge time constants for the plasma membrane of mammalian cells are characteristically in the order of nanosecond level. In conventional microsecond-duration electrical pulses, the charge of the cell plasma membrane compensates for the external electric field and protects the cell interior. However, for field magnitudes greater than 10 kV/cm, nsPEF can charge smaller intracellular structures to the electroporation threshold faster than the plasma membrane can charge and protect these structures (37, 38). The powerful ability of nsPEF to eradicate tumor has been confirmed by several studies of liver cells and animal models. Moreover, the animal models showed that blood vessels and bile ducts within or directly adjacent to the ablation zone remain undamaged. Hence, we designed this trial to explore and evaluate the feasibility, efficacy, and safety of nsPEF in the treatment of HCC.

This trial is currently recruiting patients. The first patient was enrolled on April 13, 2020. At present, the protocol is effective and 4 centers are actively recruiting patients for the trial. One hundred and eighty-three of 192 patients (95%) have been recruited. It is estimated that recruitment will be completed in December 2022. One case was showed in **Figure 5**, the tumor located in close contact with gallbladder and right portal branch, which had an obvious high risk of thermal damage with RFA or MWA. Thermal damage to adjacent non-targeted structures can result in serious complications, such as hemorrhage, biliary fistula or intestinal perforation. In addition, heat drawn away from the targeted tumor by the surrounding vessel may result in incomplete ablation. For these reasons, the patient was enrolled in the clinical trial and underwent nsPEF treatment.

Based on the preoperative images, the number, size, shape, margins, blood supply, and relationships with adjacent structures of the target tumor were determined. A reasonable arrangement of nsPEF electrodes were designed, including the number of electrodes, active tip length of electrodes, puncture path and distances between the electrodes. Because the maximum diameter of the tumor was 1.9 cm, the two-electrode configuration was selected. Electrodes were inserted along the long axis of the tumor with active tip length of 2.0 cm. The shortest puncture path was selected while avoiding damage to the surrounding important structures. After all the electrodes are precisely placed under ultrasound guidance, a test pulse at 5 kV was delivered. After the test pulse confirms adequate conductivity, nsPEF was conducted with 800 pulses, a pulse length of 300 ns, with electrocardiographic triggering. During nsPEF, the head and tail end of the active tip of the electrodes had punctuated enhancements on ultrasound, which was used to identify the orientation of the electrodes and to further verify the correct placement of the electrodes as planned. The CEUS was performed immediately after nsPEF to evaluate whether the nonenhanced area completely covers the tumor. In this case, the CEUS images after ablation showed the target tumor was covered completely by the ablation zone, and there was no evidence of local complications of biliary or vascular injury. The patient was discharged the next day after nsPEF. As with all ablation techniques, sufficient preoperative preparation, standardized operative procedures, precise positioning, use of reasonable ablation parameter settings and fine postoperative management are important for reducing the incidence of complications. Follow-up MR images on the 16 months after nsPEF revealed adequate shrinkage of the ablation zone was observed, without signs of residual tumor. As showed in this case, the pilot results of our study suggest that nsPEF is an effective and safe technique to treat HCC located close to critical structures, which considered contraindicated to thermal ablation.

Under these challenging inclusion criteria, our initial treatment course of nsPEF achieved 87% complete ablation based on polit data. No collateral thermal damage to the main bile duct or hepatic vascular structures were encountered. In addition, we found that nsPEF induced more slight muscle contraction than traditional IRE. Further analysis with long-term follow-up is required at the end of the recruitment.

The most important limitation of our protocol is its nonrandomized nature. However, nsPEF will be performed in only those patients with tumors close to the portal vein, hepatic veins, bile duct, gastrointestinal tract, or diaphragm (i.e., patients who are not suitable candidates for thermal ablation and surgery). Since these later methods would predictably fail for patients in this study, we favored a single-arm study design with an effectiveness threshold. Along with the scientific and deeper understanding for pathophysiology mechanism of HCC, recent researches have revealed the role of BRAF in HCC, which long non-coding RNA of BRAF may be another mechanism of cancer proliferation and tyrosine kinase inhibitors escape in HCC (39). Targeted therapy combinations, including BRAF pathway, may bring light in new treatment of HCC. With initial promising results of this study, further relevant studies would be useful, such as nsPEF treatment compared to targeted therapy, or nsPEF combined with multi-pathways inhibition therapy. These exploring studies may open the door for better results in the treatment of HCC.

In summary, this study is the first-in-human trial to evaluate the efficacy and safety of nsPEF in patients with HCC who are considered unsuitable for thermal ablation. The design of the trial and its primary, secondary, and exploratory endpoints have the potential to broaden our understanding of electroporation-based technologies in medicine, and provide new minimally invasive therapeutic pathways for HCC at high-risk locations.

## Data Availability Statement

The raw data supporting the conclusions of this article will be made available by the authors, without undue reservation.

## Ethics Statement

The studies involving human participants were reviewed and approved by First Affiliated Hospital of Zhejiang University, First Affiliated Hospital of Zhengzhou University, Shulan Hospital, and First Affiliated Hospital of Xinjiang Medical University. The patients/participants provided their written informed consent to participate in this study.

## Author Contributions

Conceptualization: T’AJ, XC and MX. Data curation: MX, DX, GD, ZR,WZ,QZ, TA,T’AJ and X-HC. Formal analysis: T’AJ, DX, XC and MX. Investigation: T’AJ, XC and MX. Supervision: T’AJ and XC. Original draft: MX. Review and editing: T’AJ, XC and ZR. All authors contributed to the article and approved the submitted version.

## Funding

This trial is mainly funded by Hangzhou Ruidi Biotech Ltd. Hangzhou Ruidi Biotech Ltd is a medical company that aims at researching, developing, manufacturing, selling, and renting class III medical devices. This work is also supported by Development Project of National Major Scientific Research Instrument(82027803), National Natural Science Foundation of China(81971623) and Key Project of Natural Science Foundation of Zhejiang Province(LZ20H180001). The funder was not involved in the study design, collection, analysis, interpretation of data, the writing of this article or the decision to submit it for publication.

## Conflict of Interest

The authors declare that the research was conducted in the absence of any commercial or financial relationships that could be construed as a potential conflict of interest.

## Publisher’s Note

All claims expressed in this article are solely those of the authors and do not necessarily represent those of their affiliated organizations, or those of the publisher, the editors and the reviewers. Any product that may be evaluated in this article, or claim that may be made by its manufacturer, is not guaranteed or endorsed by the publisher.
