# Supplementary material for: The Safety and Efficacy of Nanosecond Pulsed Electric Field in Patients With Hepatocellular Carcinoma: A Prospective Phase 1 Clinical Study Protocol
Source: Front Oncol. 2022 Jul 13;12:869316. doi: 10.3389/fonc.2022.869316 (PMC9328750; doi:10.3389/fonc.2022.869316)
Supplement: Supplementary file 1 [file DataSheet_1.docx]

**Supplementary**

**Appendix 1**

**Significant findings from preclinical nsPEF studies in the liver**

|  | | **Researchers** | **Year** | **nsPEF parameters** | **Experimental animals and cell line** | **Results** |  |
| --- | --- | --- | --- | --- | --- | --- | --- |
| 1 | | Qian(1) | 2020 | 20pulses, 100ns long and, 30 kV/cm, 40 kV/cm, 50 kV/cm | Four liver cancer cell lines (HCCLM3, HuH-6, Hepa 1–6, H22);  Male C57BL/6 mice | The nsPEF treatment resulted in the increased PD-L1 level and dysfunction of infiltrated CD8+ T cells in tumor tissues in vivo, indicating the long-term antitumor efficacy of nsPEF treatment. |  |
| 2 | | Yimingjiang(2) | 2020 | 1000 pulses,300 ns and 20 kV/cm | Human HCC cell lines  Hepa1–6 and C57BL-6J; mice (6–12 weeks of age) | Both nsPEF ablation and anti-PD-1 treatment induced immune cell infiltration in local tumors and modulated cytokine levels in the peripheral blood, with distinct changes in the two treatment groups. |  |
| 3 | Qian(3) | | 2020 | 300pulses, 100ns and 25 kV/cm with a rate of 1Hz | C57BL6 male mice (10 weeks old) | The nsPEF not only was a safe ablation approach but also could stimulate the regeneration of the whole liver through the activation of the HGF/c-Met pathway by upregulation of PDGF within the periablational zone |  |
| 4 | Chen(4) | | 2017 | 100ns,7kV/cm,14kV/cm,21 kV/cm | Fmale 8-weeks old C57B/6 mice | The nsPEF ablation can be applied on hepatic hydatid by inhibiting parasite growth, destructing the cyst and stimulating infections |  |
| 5 | Chen(5) | | 2017 | 40 kV/cm with 500 pulses at 1 Hz | spontaneous osteosarcomas and nude mice | Locally applied nanosecond pulsed electric field is a novel non-thermal ablation method. It can ablate the primary tumor and decrease lung metastasis as a palliative therapy for late-stage tumor. |  |
| 6 | Yin(6) | | 2016 | 12 pulses, 100 ns long, 40 kV/cm, | The SMMC7721, BEL7402 cells and High  metastatic HCC cell line HCCLM3 | The nsPEF disrupt the microdomains on the outer cellular membrane directly and increase the membrane permeabilization for PI and cisplatin. The microdomain disruption and membrane infiltration changes are caused by the mechanical force from the changes of negative cell surface charge |  |
| 7 | Nuccitelli(7) | | 2015 | 400 pulses, 100 ns and 15 kV | Male Buffalo rats (Charles  River, Cambridge,MA) and the isogenic hepatocellular carcinoma cell line,McA-RH7777 | We conclude that nanoelectroablation triggers the production of CD8+ cytotoxic T-cells resulting in the inhibition of secondary tumor growth. |  |
| 8 | Chen(8) | | 2014 | 1000pulses,100 ns and 50kV/cm with repetition rates of 1 Hz | Male Sprague Dawley Rats (250 g; Harlan (Frederick, MD or Dublin, VA) and The N1-S1 HCC cell line | NsPEFs not only eliminate N1-S1 HCC tumors, but also may induce an immuno-protective effect that defends animals against recurrences of the same cancer |  |
| 9 | Yin(9) | | 2014 | 100 ns,40 kV/cm with a rate of 0.5 Hz | Human hepatocellular carcinoma cell line SMMC7721,macrophage cell line THP1 and High metastatic HCC  cell line HCCLM3. BALB/c nude mice | The nsPEF is efficient in controlling HCC progression and reducing its metastasis. The nsPEF treatment may elicit a host immune response against tumor cells. |  |
| 10 | Chen(10) | | 2014 | 10pules,100 ns and 0–60 kV/cm | hepatocellular carcinoma cell lines HepG2, SMMC7721, Hep1-6, and HCCLM3  BALB/c nude mouse | The low dose multiple nsPEF application is more efficient than high dose single treatment in inhibiting the tumor volume in vivo, which is quite different from the dose-effect relationship in vitro. |  |
| 11 | Chen(11) | | 2012 | 30 or 100ns and strengths 33,50 and 68 kV/cm | Hepa1-6 murine hepatoma cells and female C57BL/6  mice (4 wks, ~20 g; Charles River) | The nsPEF ablation eliminated hepatocellular carcinoma tumors by targeting two therapeutic sites, apoptosis induction and inhibition of angiogenesis, both important cancer hallmarks. |  |
| 12 | Beebe(12) | | 2011 | 300 ns, 1–80 kV/cm | murine B16F10 | Using pulses with 60 or 300 ns and electric fields as high as 60 kV/cm, murine Hepa 1-6, rat N1S1 and human HepG2 HCC are readily eliminated with changes in caspase-3 activity. |  |

**Appendix 2**

**Common Terminology Criteria for Adverse Events (CTCAE) v5.0**

**Grade 1** Mild; asymptomatic or mild symptoms; clinical or diagnostic observations only; intervention not indicated.

**Grade 2** Moderate; minimal, local or noninvasive intervention indicated; limiting age-appropriate instrumental ADL*.

**Grade 3** Severe or medically significant but not immediately life-threatening; hospitalization or prolongation of hospitalization indicated; disabling; limiting self care ADL**.

**Grade 4** Life-threatening consequences; urgent intervention indicated.

**Grade 5** Death related to AE.

Activities of Daily Living (ADL) * Instrumental ADL refer to preparing meals, shopping for groceries or clothes, using the telephone, managing money, etc. **Self care ADL refer to bathing, dressing and undressing, feeding self, using the toilet, taking medications, and not bedridden.

1. Qian J, Chen T, Wu Q, Zhou L, Zhou W, Wu L, et al. Blocking exposed PD-L1 elicited by nanosecond pulsed electric field reverses dysfunction of CD8(+) T cells in liver cancer. *Cancer Lett* (2020) 495:1-11. Epub 2020/09/20. doi: 10.1016/j.canlet.2020.09.015. PubMed PMID: 32949680.

2. Yimingjiang M, Tuergan T, Chen X, Wen H, Shao Y, Zhang R, et al. Comparative Analysis of Immunoactivation by Nanosecond Pulsed Electric Fields and PD-1 Blockade in Murine Hepatocellular Carcinoma. *Anal Cell Pathol (Amst)* (2020) 2020:9582731. Epub 2020/08/18. doi: 10.1155/2020/9582731. PubMed PMID: 32802733; PubMed Central PMCID: PMCPMC7416239.

3. Qian J, Liu J, Hong L, Lu H, Guo D, Liu Z, et al. Upregulation of PDGF Mediates Robust Liver Regeneration after Nanosecond Pulsed Electric Field Ablation by Promoting the HGF/c-Met Pathway. *Biomed Res Int* (2020) 2020:3635787. Epub 2020/04/08. doi: 10.1155/2020/3635787. PubMed PMID: 32258116; PubMed Central PMCID: PMCPMC7097769.

4. Chen X, Zhang R, Aji T, Shao Y, Chen Y, Wen H. Novel Interventional Management of Hepatic Hydatid Cyst with Nanosecond Pulses on Experimental Mouse Model. *Sci Rep* (2017) 7(1):4491. Epub 2017/07/05. doi: 10.1038/s41598-017-04873-5. PubMed PMID: 28674451; PubMed Central PMCID: PMCPMC5495767.

5. Chen X, Chen Y, Jiang J, Wu L, Yin S, Miao X, et al. Nano-pulse stimulation (NPS) ablate tumors and inhibit lung metastasis on both canine spontaneous osteosarcoma and murine transplanted hepatocellular carcinoma with high metastatic potential. *Oncotarget* (2017) 8(27):44032-9. PubMed PMID: 28476039.

6. Yin S, Chen X, Xie H, Zhou L, Guo D, Xu Y, et al. Nanosecond pulsed electric field (nsPEF) enhance cytotoxicity of cisplatin to hepatocellular cells by microdomain disruption on plasma membrane. *Exp Cell Res* (2016) 346(2):233-40. Epub 2016/07/05. doi: 10.1016/j.yexcr.2016.06.018. PubMed PMID: 27375200.

7. Nuccitelli R, Berridge JC, Mallon Z, Kreis M, Athos B, Nuccitelli P. Nanoelectroablation of Murine Tumors Triggers a CD8-Dependent Inhibition of Secondary Tumor Growth. *PLoS One* (2015) 10(7):e0134364. PubMed PMID: 26231031.

8. Chen R, Sain NM, Harlow KT, Chen YJ, Shires PK, Heller R, et al. A protective effect after clearance of orthotopic rat hepatocellular carcinoma by nanosecond pulsed electric fields. *Eur J Cancer* (2014) 50(15):2705-13. Epub 2014/08/02. doi: 10.1016/j.ejca.2014.07.006. PubMed PMID: 25081978.

9. Yin S, Chen X, Hu C, Zhang X, Hu Z, Yu J, et al. Nanosecond pulsed electric field (nsPEF) treatment for hepatocellular carcinoma: a novel locoregional ablation decreasing lung metastasis. *Cancer Lett* (2014) 346(2):285-91. Epub 2014/01/28. doi: 10.1016/j.canlet.2014.01.009. PubMed PMID: 24462824.

10. Chen X, Yin S, Hu C, Chen X, Jiang K, Ye S, et al. Comparative study of nanosecond electric fields in vitro and in vivo on hepatocellular carcinoma indicate macrophage infiltration contribute to tumor ablation in vivo. *PLoS One* (2014) 9(1):e86421. PubMed PMID: 24475118.

11. Chen X, Zhuang J, Kolb JF, Schoenbach KH, Beebe SJ. Long term survival of mice with hepatocellular carcinoma after pulse power ablation with nanosecond pulsed electric fields. *Technol Cancer Res Treat* (2012) 11(1):83-93. Epub 2011/12/21. doi: 10.7785/tcrt.2012.500237. PubMed PMID: 22181334.

12. Beebe SJ, Chen X, Liu JA, Schoenbach KH. Nanosecond pulsed electric field ablation of hepatocellular carcinoma. *Annu Int Conf IEEE Eng Med Biol Soc* (2011) 2011:6861-5. Epub 2012/01/19. doi: 10.1109/IEMBS.2011.6091692. PubMed PMID: 22255915.
